# White matter hyperintensities associated with impulse control disorders in Parkinson’s Disease

**DOI:** 10.1038/s41598-023-37054-8

**Published:** 2023-06-30

**Authors:** Gabriella Hernadi, Gabor Perlaki, Marton Kovacs, David Pinter, Gergely Orsi, Jozsef Janszky, Norbert Kovacs

**Affiliations:** 1 Pecs Diagnostic Centre, Pecs, Hungary; 2 ELKH-PTE Clinical Neuroscience MR Research Group, Pecs, Hungary; 3 Department of Neurology, Medical School, University of Pecs, Pecs, Hungary; 4 Department of Neurosurgery, Medical School, University of Pecs, Pecs, Hungary

**Keywords:** Neurology, Parkinson's disease

## Abstract

Impulse control disorders (ICDs) in Parkinson's disease (PD) are increasingly recognized as clinically significant non-motor features that potentially impair the quality of life. White matter hyperintensities (WMHs), detected by magnetic resonance imaging, are frequently observed in PD and can be associated with both motor- and certain non-motor symptoms. Given the limited number of non-motor features studied in this context, our aim was to reveal the potential association between the severity of WMHs and ICDs in PD. Fluid-attenuated inversion recovery magnetic resonance images were retrospectively evaluated in 70 patients with PD (48 males; 59.3 ± 10.1 years). The severity of WMHs was assessed by Fazekas scores and by the volume and number of supratentorial WMHs. ICDs were evaluated using the modified Minnesota Impulsive Disorders Interview. Significant interaction between age and the severity of WMHs was present for ICDs. In our younger patients (< 60.5 years), severity of WMHs was positively associated with ICDs (*p* = 0.004, *p* = 0.021, *p* < 0.001 and *p* < 0.001, respectively for periventricular white matter and total Fazekas scores and the volume and number of WMHs). Our study supports the hypothesis that WMHs of presumed vascular origin may contribute to ICDs in PD. Future prospective studies are needed to assess the prognostic relevance of this finding.

## Introduction

In addition to the cardinal motor symptoms of Parkinson’s Disease (PD), such as bradykinesia, rigidity, resting tremor and disturbances of gait and balance, impulse control disorders (ICDs) and related behaviors (ICDRBs) are increasingly recognized clinically significant non-motor features of the disorder^1^. The four major symptoms of ICD in PD include pathological gambling, compulsive buying, hypersexuality and binge eating^2^. Besides the four main ICDs, other impulsive-compulsive behaviors—such as dopamine dysregulation syndrome, punding, hobbyism and walkabout—have been reported to occur in PD and often termed as ICDRBs^1,3,4^. The prevalence of ICD in PD can be more than 25%^5^ and in line with the suggestion of an earlier narrative review about higher prevalence of ICDs in PD^6^, a recent meta-analysis has also demonstrated a greater risk for ICDs in patients with PD compared to healthy controls^7^. Moreover, ICD is possible much more common in PD than previously reported, because the detection at an early phase is quite challenging and standard screening methods may be imperfect^8^. However, the increased rate of ICDs in PD is not necessarily correct in general, especially in case of untreated de novo patients, where ICDs and related behavior symptoms occur at a similar rate to healthy controls^3,9^. These results support an idea that not the disease itself, but other important factors lead to the development of ICDs in PD^3^.

It is relatively well established that antiparkinsonian medications, especially the use of dopamine agonists (DAs) are strongly associated with ICDs^1,10,11^. The association between DA usage and ICDs is not unique to PD, as it has already been demonstrated in other clinical conditions (e.g. fibromyalgia or restless legs syndrome)^1^. However, on the other hand, even drug naïve, de novo patients may be positive for ICDs^3,9^, making it evident that ICDs in PD are multifactorial^7^ and not a simple side-effect of antiparkinsonian medications. Several additional key factors contributing to ICDs in PD have been suggested, including demographic (e.g. younger age, being unmarried, male gender, living in the U.S.), disease-related (e.g. younger age at PD onset, longer disease duration), addictive (e.g. smoking, family history of gambling problems), personality-related (e.g. impulsivity or novelty seeking), psychiatric (e.g. depression) and genetic factors among others^1,10,12,13^.

To explore cerebral changes associated with ICD in PD and to better understand the underlying anatomical and functional mechanisms, several neuroimaging studies were carried out, including structural and functional MRI, PET, and SPECT studies. Since the results are quite inconsistent across the studies, reviews and meta-analytic articles tried to integrate findings and recognize consistencies^14–17^. While a recent meta-analysis suggested significant cortical thinning in the right precentral, inferior- and middle frontal gyri, the left superior frontal and cingulate gyri of PD patients with ICD, this conclusion was based on only three studies^15^ and other reviews revealed that morphometric findings are rather too inconsistent to reach any firm conclusion about the structural brain changes associated with ICD in PD^14,16,17^. A recent review of brain activity studies (including fMRI, PET and SPECT studies) suggested the involvement of ventral striatum, orbitofrontal cortex and anterior cingulate cortex (ACC), with the role of ventral striatum and ACC confirmed by activation likelihood estimation (ALE) meta-analysis as well^16^. The involvement of the default mode-, the salience- and the central-executive networks also seems consistent as indicated by resting-state fMRI studies^14,18,19^. Additionally, a few diffusion MRI studies are also available in the literature. Zadeh et al. reported disrupted connectivity in numerous white matter tracts of drug-naïve PD patients with ICDs including the corticothalamic, corticopontine and corticospinal tracts as well as the middle- and superior cerebellar peduncles, inferior longitudinal fasciculus and the cingulum^20^. Increased diffusivity and decreased fractional anisotropy—suggesting reduced white matter tract integrity—were also presented in PD patients with impulsive–compulsive behaviors by two studies with partially overlapping samples^21,22^. Interestingly, others using diffusion kurtosis imaging (DKI) and magnetization transfer imaging suggested that white matter microstructural changes compared to healthy controls might be less prominent in PD patients with impulse control behaviors than in patients without such behaviors^23^. This finding is in line with a diffusion tensor imaging (DTI) study indicating relatively preserved white matter integrity in PD patients with ICD compared to patients without ICD^24^. Taken together, it seems quite obvious that changes in white matter microstructure and white matter connectivity also play a crucial role in the development of PD-related ICD, therefore white matter should be an important target for future studies. However, the difficulties in applying these microstructural findings to clinical practice should be noted. DTI or more complex diffusion approaches such as DKI are usually still not part of clinical routine; the main reasons limiting their routine use probably include the followings: time-consuming nature of the required post-processing and subsequent statistical analyses, uncertain diagnostic significance, challenges of projecting group-level findings to individual level and interpretation of the data at a subject level^25,26^. Moreover, the subtle microstructural alterations should be visually detectable as the evaluation with diagnostic purpose is almost exclusively based on qualitative visual inspection^25^.

In contrast to white matter microstructure, white matter hyperintensities (WMHs) can be easily assessed in a clinical setting and they are commonly observed in routine MRI scans of older adults, including PD patients^27–30^. Although the risk for incidental WMHs is increased with advancing age, they should not be overlooked or generally considered as inherent “silent” consequences of normal aging; the clinical importance was reviewed by Wardlaw et al.^31^. The prevalence of WMHs is highly variable and according to a recent systematic review and meta-analysis, WMHs are not necessarily more prevalent or severe in PD compared to controls^28^. However, besides the studies demonstrating that WMHs are associated with motor symptoms and cognitive impairment, some studies also suggest the relationship between non-motor symptoms of PD and WMHs^27,29,30^. Based on this, one can hypothesize that WMHs might be also related to development of ICDs. Therefore, the aim of the current retrospective study was to investigate the association between WMHs and ICDs in PD.

## Materials and methods

### Subjects

Data for this retrospective study were collected from PD patients treated at the University of Pécs who underwent detailed neuropsychiatric assessment and routine MRI between December 2014 and July 2021 as a part of a thorough examination before their deep brain stimulation (DBS) surgery. All patients fulfilled the UK PD Society Brain Bank Diagnostic Criteria and had no significant cognitive impairment or psychiatric problems. Of the 83 patients in this group, 13 were excluded before any further analysis due to missing ICD data (n = 1), left-handedness (n = 1), insufficient imaging data (n = 1), and the suspicion of following conditions based on the MR images: radiologically isolated syndrome (n = 1), presence of cortical, basal ganglia or pons lesions (n = 5), cavernoma (n = 1), developmental venous anomaly (n = 2), marked thickening of the skull combined with olfactory meningioma (n = 1). The final sample included 70 subjects (48 males; mean age: 59.3 ± 10.1, range: 38–79 years). Each subject gave written informed consent in accordance with the ethical approval of the Institutional and Regional Ethical Board of the University of Pécs (7069-PTE 2018) and performed in accordance with the Declaration of Helsinki.

### Image acquisition and evaluation

Routine brain MRI at 1.5 or 3 T Siemens scanner was performed in all subjects. Coronal fluid-attenuated inversion recovery (FLAIR) images (TR: 5000–9430 ms; TE: 85–95 ms; TI: 1800–2790 ms; in-plane resolution of 0.72 × 0.72–0.88 × 0.88 mm^2^; slice thickness: 4–5 mm; gap: 20–30%; number of slices: 33–43; receiver bandwidth = 181–287 Hz/pixel) were used for grading severity of supratentorial WMHs by an experienced neuroradiologist (G.H.) blinded to any clinical data. Images were visually inspected to confirm appropriate image quality for WMH assessment. Depending on the size and confluence of the lesions, deep- and periventricular WMHs were visually rated according to the 4-point scale (i.e. 0–3) suggested by Fazekas et al.^32^; hereafter referred to as deep white matter (DWM) and periventricular white matter (PVWM) Fazekas scores. When the rater was unable to decide between two grades (e.g. grade = 1 vs. grade = 2), the average being halfway between them was taken (i.e. grade = 1.5). Total Fazekas WMH score was created by taking the maximum of DWM- and PVWM Fazekas scores.

Supratentorial WMHs were also assessed by manual segmentation on the coronal FLAIR images using 3D Slicer (4.10.2 r28257). The total volume and number of supratentorial WMHs were extracted for each subject using the *fslstats* tool in FSL (https://fsl.fmrib.ox.ac.uk/fsl/fslwiki/Fslutils).

### Demographic and clinical characteristics

ICD was assessed using the modified Minnesota Impulsive Disorders Interview (mMIDI)^33^, including 5 modules measuring different impulsive-compulsive behaviors such as compulsive buying, compulsive gambling, compulsive sexual behavior, compulsive eating, and punding behavior. In case of an affirmative response to the gateway question of a module, additional follow-up questions were presented about the actual behavior. The Total mMIDI score (ranging 0–56) was calculated for each subject with higher score indicating more severe ICD.

To assess disease severity, the Movement Disorder Society-sponsored Unified Parkinson’s Disease Rating Scale (MDS-UPDRS), the Hoehn–Yahr stage (HYS) and disease duration were used^34–36^. Because MDS-UPDRS Part I also contains an item about the features of dopamine dysregulation syndrome (i.e. an ICD related behavior)^34^, we also calculated the modified version of MDS-UPDRS Part I (mMDS-UPDRS Part I) and MDS-UPDRS Total (mMDS-UPDRS Total) scores by excluding this item (i.e. item 1.6) from the calculation. For the better characterization of patients, additional disease-specific data (e.g. age at PD diagnosis, type of PD and medication usages/doses) and non-motor features such as apathy, depression or anxiety (i.e. Lille Apathy Rating Scale, LARS^37^; Montgomery-Asberg Depression Rating Scale, MADRS^38^; and Parkinson Anxiety Scale, PAS^39^) were also assessed in addition to the demographic data (i.e. age, sex, and level of education). Demographic data and clinical characteristics are presented in Table 1.

**Table 1:** Demographic and clinical characteristics.

	Whole patient group (n = 70)	Younger group (n = 35)	Older group (n = 35)	*p*-value
Sex (M/F)	48/22	26/9	22/13	0.440^b^
Age (years)	59.3 ± 10.1 [38–79]; 60.5 (51.4–67.3)	51.2 ± 6.4 [38–60]; 51.7 (44.7–56.5)	67.4 ± 5.5 [61–79]; 67.1 (62.2–71.8)	** < 0.001**^**c**^
Education (years)	13.8 ± 3.0 [8–21]; 12.5 (11.0–16.0)	12.9 ± 2.7 [8–20]; 12.0 (11.0–14.0)	14.8 ± 3.0 [8–21]; 16.0 (12.0–16.0)	**0.006**^**c**^
Disease severity				
HYS 1	2 (2.9%)	2 (5.7%)	0 (0%)	0.060^c^
HYS 2	52 (74.3%)	28 (80%)	24 (68.6%)	
HYS 3	11 (15.7%)	3 (8.6%)	8 (22.9%)	
HYS 4	4 (5.7%)	2 (5.7%)	2 (5.7%)	
HYS 5	1 (1.4%)	0 (0%)	1 (2.9%)	
Disease duration (years)	8.3 ± 4.4 [1–20]; 8.0 (5.0–10.0)	7.9 ± 3.4 [3–20]; 7.0 (5.0–10.0)	8.7 ± 5.2 [1–20]; 9.0 (4.0–10.0)	0.697^c^
Age at PD diagnosis (years)	51.0 ± 10.5 [27–75]; 50.5 (44.0–58.0)	43.3 ± 6.9 [27–56]; 45.0 (37.0–49.0)	58.7 ± 7.5 [41–75]; 58.0 (55.0–61.0)	** < 0.001**^**c**^
Disease subtype				
Rigid-akinetic	36 cases	18 cases	18 cases	1.0^b^
Tremor-dominant	15 cases	8 cases	7 cases	
Mixed	19 cases	9 cases	10 cases	
Levodopa usage	67 (95.7%)	34 (97.1%)	33 (94.3%)	1.0^b^
DA usage	46 (65.7%)	21 (60.0%)	25 (71.4%)	0.450^b^
MAOI usage	24 (34.3%)	15 (42.9%)	9 (25.7%)	0.208^b^
COMTI usage	53 (75.7%)	30 (85.7%)	23 (65.7%)	0.093^b^
ACh usage	2 (2.9%)	1 (2.9%)	1 (2.9%)	1.0^b^
Levodopa LED (mg)	879 ± 564 [0–2795]; 790 (569–1148)	917 ± 588 [0–2730]; 813 (500–1300)	841 ± 546 [0–2795]; 780 (585–1040)	0.638^c^
DA LED (mg)	245 ± 246 [0–923]; 180 (0–413)	207 ± 219 [0–769]; 160 (0–400)	283 ± 268 [0–923]; 240 (0–480)	0.267^c^
LEDD (mg)	1241 ± 643 [0–3575]; 1215 (809–1586)	1246 ± 599 [0–2850]; 1240 (845–1530)	1235 ± 694 [0–3575]; 1215 (750–1724)	0.882^c^
MDS-UPDRS I	12.5 ± 5.4 [1-26]; 12.0 (8.8–16.0)	12.0 ± 5.4 [1–23]; 11.0 (9.0–17.0)	12.9 ± 5.4 [3–26]; 13.0 (8.0–16.0)	0.529^c^
mMDS-UPDRS I	12.2 ± 5.2 [1–25]; 12.0 (8.0–16.0)	11.7 ± 5.1 [1–21]; 11.0 (8.0–16.0)	12.7 ± 5.3 [3–25]; 12.0 (8.0–16.0)	0.510^c^
MDS-UPDRS II	16.4 ± 7.2 [3–33]; 15.0 (12.0–22.0)	16.9 ± 6.5 [5–30]; 17.0 (12.0–23.0)	15.9 ± 7.8 [3–33]; 15.0 (10.0–21.0)	0.507^c^
MDS-UPDRS III	26.2 ± 14.2 [8–80]; 22.5 (15.0–32.0)	23.9 ± 14.0 [8–59]; 19.0 (14.0–30.0)	28.6 ± 14.2 [9–80]; 26.0 (21.0–36.0)	0.060^c^
MDS-UPDRS IV	6.3 ± 3.7 [0–16]; 6.5 (4.0–9.0)	6.9 ± 3.7 [0–16]; 7.0 (5.0–9.0)	5.7 ± 3.6 [0–12]; 5.0 (2.0–9.0)	0.181^c^
MDS-UPDRS Total	61.4 ± 23.8 [24–133]; 54.5 (44.0–78.3)	59.7 ± 23.7 [28–116]; 53.0 (42.0–78.0)	63.1 ± 24.2 [24–133]; 59.0 (45.0–79.0)	0.519^c^
mMDS-UPDRS Total	61.1 ± 23.6 [24–133]; 54.0 (43.8–77.5)	59.3 ± 23.3 [28–114]; 53.0 (42.0–77.0)	62.8 ± 24.1 [24–133]; 59.0 (45.0–79.0)	0.496^c^
LARS^a^	− 26.7 ± 5.7 [− 36; − 6]; − 28.0 (− 30.5; − 24.0)	− 26.4 ± 6.4 [− 36; − 6]; − 28.0 (− 31.0; − 25.0)	− 26.9 ± 5.0 [− 34; − 9]; − 28.0 (− 30.3; − 23.8)	0.950^c^
MADRS	11.0 ± 5.8 [1–26]; 10.0 (7.0–15.0)	11.4 ± 6.1 [1–26]; 11.0 (7.0–16.0)	10.6 ± 5.5 [2–24]; 10.0 (7.0–15.0)	0.572^c^
PAS	12.6 ± 6.7 [0–29]; 12.0 (7.0–16.3)	14.3 ± 5.6 [5–28]; 14.0 (10.0–18.0)	10.9 ± 7.3 [0–29]; 11.0 (5.0–16.0)	**0.048**^**c**^
Total mMIDI	3.1 ± 4.5 [0–20]; 0.0 (0.0–6.0)	3.9 ± 5.5 [0–20]; 0.0 (0.0–7.0)	2.3 ± 3.2 [0–9]; 0.0 (0.0–6.0)	0.383^c^
Total mMIDI > 0	30 (42.9%)	16 (45.7%)	14 (40.0%)	0.809^b^
mMIDI Buying > 0	8 (11.4%)	3 (8.6%)	5 (14.3%)	0.710^b^
mMIDI Gambling > 0	5 (7.1%)	5 (14.3%)	0 (0.0%)	0.054^b^
mMIDI Sexuality > 0	3 (4.3%)	3 (8.6%)	0 (0.0%)	0.239^b^
mMIDI Eating > 0	7 (10.0%)	3 (8.6%)	4 (11.4%)	1.0^b^
mMIDI Punding > 0	21 (30.0%)	11 (31.4%)	10 (28.6%)	1.0^b^
Fazekas PVWM				
Grade = 0	43 (61.4%)	25 (71.4%)	18 (51.4%)	**0.041**^**c**^
0.5 ≤ grade ≤ 1	26 (37.1%)	10 (28.6%)	16 (45.7%)	
Grade ≥ 1.5	1 (1.4%)	0 (0%)	1 (2.9%)	
Fazekas DWM				
Grade = 0	31 (44.3%)	20 (57.1%)	11 (31.4%)	0.072^c^
0.5 ≤ grade ≤ 1	35 (50.0%)	14 (40.0%)	21 (60%)	
Grade ≥ 1.5	4 (5.7%)	1 (2.9%)	3 (8.6%)	
Total Fazekas score				
Grade = 0	26 (37.1%)	19 (54.3%)	7 (20.0%)	**0.010**^**c**^
0.5 ≤ grade ≤ 1	40 (57.1%)	15 (42.9%)	25 (71.4%)	
Grade ≥ 1.5	4 (5.7%)	1 (2.9%)	3 (8.6%)	
Volume of WMHs (mm^3^)	2156 ± 6443 [0–51265]; 405 (21–1987)	945 ± 1668 [0–8314]; 165 (0–1285)	3367 ± 8857 [0–51265]; 960 (177–2748)	**0.012**^**c**^
Number of WMHs	14.9 ± 20.6 [0–88]; 6.5 (1.0–20.3)	11.1 ± 17.0 [0–60]; 2.0 (0.0–15.0)	18.7 ± 23.3 [0–88]; 8.0 (3.0–23.0)	**0.026**^**c**^

Values are given as mean ± SD [range]; median (interquartile range).

*M* Male, *F* Female, *HYS* Hoehn-Yahr stage, *PD* Parkinson’s disease, *DA* Dopamine agonist, *MAOI* Monoamine oxidase inhibitor, *COMTI* Catechol-O-methyltransferase inhibitor, *Ach* Anticholinergic, *Levodopa LED* Levodopa equivalent dose for levodopa, *DA LED* Levodopa equivalent dose for dopamine agonists, *LEDD* levodopa equivalent daily dose, *MDS-UPDRS* Movement Disorder Society-sponsored Unified Parkinson’s Disease Rating Scale, *mMDS-UPDRS I and mMDS-UPDRS Total* scores modified by excluding the item about features of dopamine dysregulation syndrome from the calculation, *LARS* Lille Apathy Rating Scale, *MADRS* Montgomery-Asberg Depression Rating Scale, *PAS* Parkinson Anxiety Scale, *mMIDI* modified Minnesota Impulsive Disorders Interview, *Fazekas PVWM* Fazekas score for periventricular white matter, *Fazekas DWM* Fazekas score for deep white matter, *Total Fazekas score* maximum of Fazekas PVWM and Fazekas DWM, *WMHs* white matter hyperintensities.

^a^One subject had missing data (i.e. whole patient group n = 69; older group = n = 34).

^b^Fischer's Exact Test.

^c^Mann-Whitney U test.

*P*-values are given for comparison of younger and older patient groups. *P*-values < 0.05 are shown in bold.

### Statistical analysis

Statistical analyses were performed using IBM SPSS Statistics for Windows, Version 25.0 (IBM Corp., Armonk, NY, USA). Our initial general linear model, with Total mMIDI score as dependent and age and number of WMHs as independent variables, indicated a strong interaction effect between age and severity of WMHs (*p* = 2∙10^–5^). Substitution of the number of WMHs with other variables in this initial model showed that there are additional interaction effects between age and a number of variables such as MDS-UPDRS Total, MDS-UPDRS Part I, Part II, Part III, mMDS-UPDRS Total and mMDS-UPDRS Part I scores, years of education, levodopa equivalent dose for dopamine agonists (DA LED), volume of WMHs, PVWM-, DWM- and Total Fazekas scores (*p*-value between: 8∙10^–4^ and 0.046). To address this problem, our patients were divided into two groups based on the median split of age (older group aged >  = 60.5 years and younger group).

Clinical and demographic characteristics were compared between younger and older patient groups using Mann–Whitney U test for ordinal or continuous data and Fischer’s exact test for categorical variables. Spearman’s rank correlation was used to correlate the Total mMIDI score with other clinical/demographic data or calculate correlations between any pair of continuous/ordinal variables. Correlation coefficient (Spearman’s rho) was interpreted as weak (0–0.299), moderate (0.300–0.599) and strong (0.600–1.000) associations.

Mann–Whitney U or Kruskall-Wallis H (if independent variable had more than 2 categories) tests were also conducted to assess differences in Total mMIDI score across different clinical/demographic categories.

In order to determine the independent effects of the severity of WMHs on ICD and to avoid overlooking some relevant predictors/influencing factors of ICD, a stepwise multiple linear regression analysis with Total mMIDI score as dependent variable was performed (Entry: *p* < 0.05; Removal: *p* > 0.1). This analysis was only performed for the younger group, where the initial correlations indicated a significant effect of WMHs at all. Variables for which we found associations with ICD in our younger group at *p* < 0.1 (see Table 2) were entered into this stepwise model, and only the modified versions of Total and Part I MDS-UPDRS scores were used as we did not want to regress out any ICD-related variance from the dependent variable. The assumptions of multiple linear regression were assessed for the final model by checking for independence of errors, linearity, homoscedasticity, multicollinearity, outliers, and normality assumptions of the residuals.

**Table 2:** Associations of demographic and clinical charachteristics with Total mMIDI score.

Associations with Total mMIDI score	Whole patient group (n = 70)		Younger group (n = 35)		Older group (n = 35)	
	rho	*p*-value	rho	*p*-value	rho	*p*-value
Sex	n.a	0.437^b^	n.a	0.391^b^	n.a	**0.022**^**b**^ (F > M)
Age (years)	− 0.110	0.366^c^	0.078	0.654^c^	− 0.173	0.320^c^
Education (years)	− 0.070	0.565^c^	0.247	0.152^c^	− 0.351	**0.038**^**c**^
HYS	0.146	0.228^c^	0.185	0.287^c^	0.153	0.381^c^
Disease duration (years)	0.177	0.142^c^	0.261	0.130^c^	0.173	0.322^c^
Age at PD diagnosis (years)	− 0.094	0.439^c^	0.047	0.788^c^	− 0.193	0.265^c^
Disease subtype	n.a	0.712^d,e^	n.a	0.708^d,e^	n.a	0.073^d,e^
Levodopa usage	n.a	0.260^b^	n.a	0.829^b^	n.a	0.491^b^
DA usage	n.a	0.051^b^	n.a	0.108^b^	n.a	0.203^b^
MAOI usage	n.a	0.317^b^	n.a	0.335^b^	n.a	0.503^b^
COMTI usage	n.a	0.071^b^	n.a	0.186^b^	n.a	0.346^b^
ACh usage	n.a	0.467^b^	n.a	0.829^b^	n.a	0.914^b^
Levodopa LED (mg)	0.170	0.160^c^	0.147	0.401^c^	0.165	0.343^c^
DA LED (mg)	0.301	**0.011**^**c**^	0.389	**0.021**^**c**^	0.258	0.134^c^
LEDD (mg)	0.297	**0.013**^**c**^	0.302	0.078^c^	0.291	0.090^c^
MDS-UPDRS I	0.426	** < 0.001**^**c**^	0.535	** < 0.001**^**c**^	0.315	0.065^c^
mMDS-UPDRS I	0.394	** < 0.001**^**c**^	0.518	**0.001**^**c**^	0.270	0.116^c^
MDS-UPDRS II	0.331	**0.005**^**c**^	0.565	** < 0.001**^**c**^	0.068	0.697^c^
MDS-UPDRS III	0.033	0.785^c^	0.167	0.339^c^	− 0.145	0.405^c^
MDS-UPDRS IV	0.174	0.149^c^	0.074	0.673^c^	0.290	0.091^c^
MDS-UPDRS Total	0.272	**0.023**^**c**^	0.465	**0.005**^**c**^	0.061	0.726^c^
mMDS-UPDRS Total	0.268	**0.025**^**c**^	0.458	**0.006**^**c**^	0.064	0.714^c^
LARS^a^	− 0.055	0.656^c^	− 0.040	0.820^c^	− 0.089	0.617^c^
MADRS	0.271	**0.023**^**c**^	0.308	0.072^c^	0.251	0.146^c^
PAS	0.259	**0.030**^**c**^	0.309	0.071^c^	0.149	0.393^c^
Fazekas PVWM	0.021	0.861^c^	0.479	**0.004**^**c**^	− 0.312	0.068^c^
Fazekas DWM	0.027	0.825^c^	0.316	0.064^c^	− 0.299	0.081^c^
Total Fazekas score	0.065	0.590^c^	0.388	**0.021**^**c**^	− 0.266	0.122^c^
Volume of WMHs (mm^3^)	0.172	0.154^c^	0.599	** < 0.001**^**c**^	− 0.306	0.074^c^
Number of WMHs	0.182	0.132^c^	0.569	** < 0.001**^**c**^	− 0.271	0.115^c^

*F* female, *M* Male, *F* > *M* Mean rank assessed by the Mann–Whitney U test is greater for females, *rho* Spearman’s correlation coefficient, *n.a.* not applicable, *HYS* Hoehn-Yahr stage, *PD* Parkinson’s disease, *Disease subtype* Rigid-akinetic, Tremor-dominant or Mixed, *DA* Dopamine agonist, *MAOI* Monoamine oxidase inhibitor, *COMTI* Catechol-O-methyltransferase inhibitor, *ACh* Anticholinergic, *Levodopa LED* Levodopa equivalent dose for levodopa, *DA LED* Levodopa equivalent dose for dopamine agonists, *LEDD* levodopa equivalent daily dose, *MDS-UPDRS* Movement Disorder Society-sponsored Unified Parkinson’s Disease Rating Scale, *mMDS-UPDRS I and mMDS-UPDRS Total* scores modified by excluding the item about features of dopamine dysregulation syndrome from the calculation, *LARS* Lille Apathy Rating Scale, *MADRS* Montgomery-Asberg Depression Rating, *PAS* Parkinson Anxiety Scale, *mMIDI* modified Minnesota Impulsive Disorders Interview, *Fazekas PVWM* Fazekas score for periventricular white matter, *Fazekas DWM* Fazekas score for deep white matter, *Total Fazekas score* maximum of Fazekas PVWM and Fazekas DWM, *WMHs* white matter hyperintensities.

^a^One subject had missing data (i.e. whole patient group n = 69; older group = n = 34).

^b^Mann-Whitney U test.

^c^Spearman’s correlation.

^d^Kruskall-Wallis test. ^e^asymptotic *p*-value.

*P*-values are given for associations of clinical/demographic data with Total mMIDI score. *P*-values < 0.05 are shown in bold.

## Results

Based on the Total mMIDI score, 30 of the 70 PD patients (42.9%) had a score greater than zero, suggesting some ICD. The rate of patients with nonzero Total mMIDI score was not different between younger and older patient subgroups (45.7% vs. 40.0%, respectively, *p* = 0.809, Table 1). Education years, age at PD diagnosis, and WMH severity (as indicated by PVWM- and Total Fazekas scores as well as volume and number of supratentorial WMHs) were all higher, while anxiety was lower in our older patient group. No other clinical or demographic characteristics were different between the two age-specific subgroups (Table 1).

Using Spearman’s correlations on all patients (n = 70), the Total mMIDI score showed moderate positive associations with MDS-UPDRS Part I, MDS-UPDRS Part II, mMDS-UPDRS Part I (rho = 0.426, *p* < 0.001, rho = 0.331, *p* = 0.005 and rho = 0.394, *p* < 0.001, respectively), and DA LED (rho = 0.301, *p* = 0.011); Table 2. Weak positive associations were also present with levodopa equivalent daily dose (LEDD) (rho = 0.297, *p* = 0.013), MDS-UPDRS Total, mMDS-UPDRS Total (rho = 0.272, *p* = 0.023 and rho = 0.268, *p* = 0.025, respectively), anxiety (PAS: rho = 0.259, *p* = 0.030), and depression (MADRS: rho = 0.271, *p* = 0.023); Table 2. The significance pattern was similar for our younger subgroup, except that WMH severity (as assessed by PVWM- and Total Fazekas scores as well as the volume and number of supratentorial WMHs) became significantly positively correlated with ICD (*p* = 0.004, *p* = 0.021, *p* < 0.001 and *p* < 0.001, respectively, Table 2) and only trend-like positive associations were observed for LEDD (*p* = 0.078), anxiety (*p* = 0.071) and depression (MADRS: *p* = 0.072). In our older subgroup, only education years and sex were found to be associated with ICD (*p* = 0.038 and *p* = 0.022, respectively).

**Figure 1:**
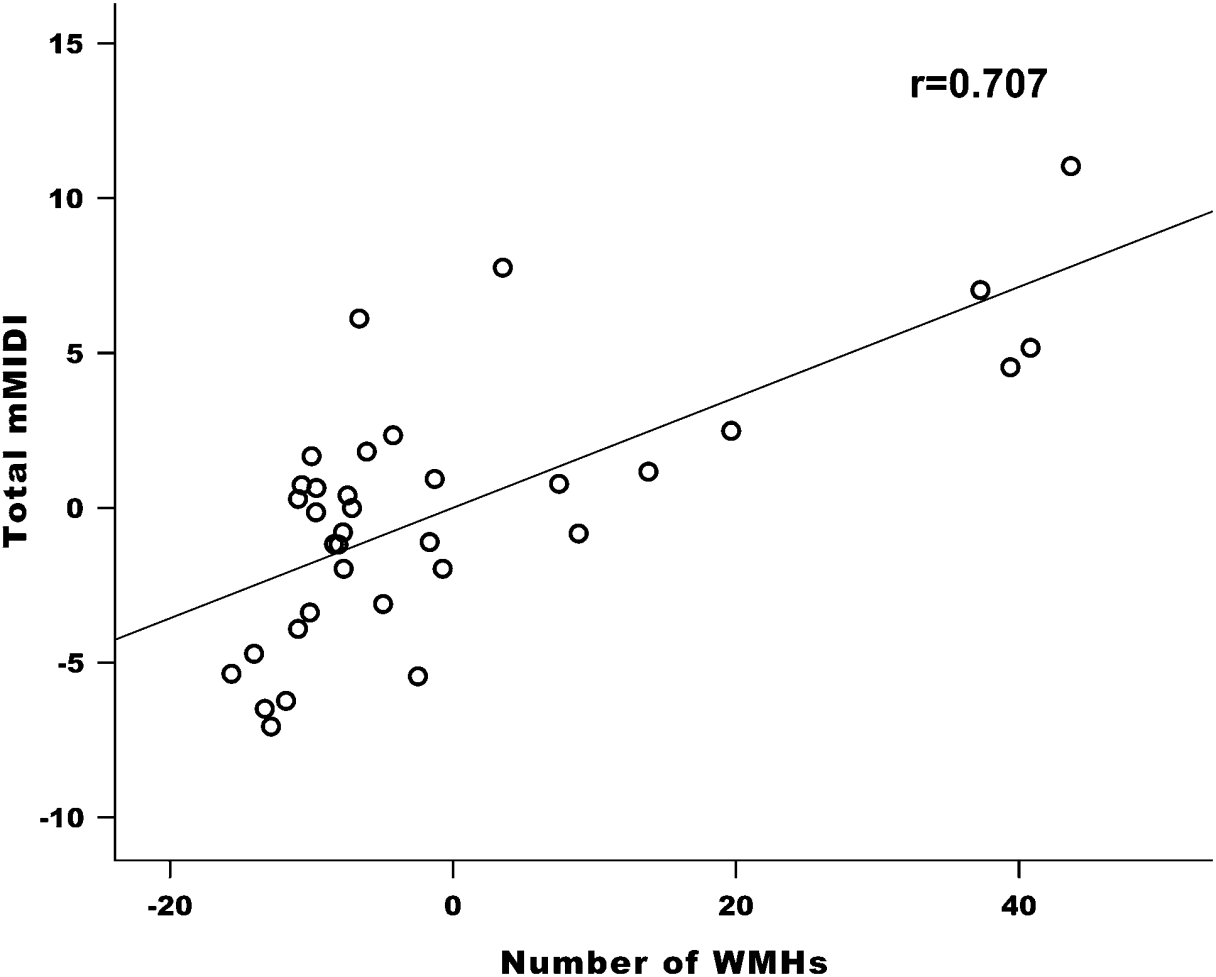
Association between the number white matter hyperintensities and impulse control disorders. The partial regression plot derived from multiple linear regression analysis demonstrates the positive relationship between the number of white matter hyperintensities (WMHs) and Total Minnesota Impulsive Disorders Interview (Total mMIDI) score after controlling for Movement Disorder Society-sponsored Unified Parkinson’s Disease Rating Scale (MDS-UPDRS) Part II and levodopa equivalent dose for dopamine agonists (DA LED). Linear correlation coefficient (r) is also presented.

Focusing on our younger group, the number of WMHs (Fig. 1), MDS-UPDRS Part II, and DA LED were selected by stepwise method as independent variables of the final multiple linear regression model and all of them were indicated as significant positive predictors of Total mMIDI score (*p* = 4·10^–6^, *p* = 3·10^–4^ and *p* = 0.005, respectively). There were no significant interactions between any pairs of the three independent variables.

## Discussion

ICDs are increasingly recognized in PD and they can significantly impair the quality of life, adversely affect caregiver and/or family burden and sometimes result in devastating consequences (e.g. social, occupational, medical, legal or financial problems)^8,40^. However, the exact pathogenesis of ICDs in PD remains unknown and there is a marked clinical need to identify further ICD-associated factors to aid early recognition of the symptoms and identification of risk groups. It has been relatively well established that white matter lesions have negative impact not only on motor symptoms but also on non-motor symptoms of PD, including cognitive impairment, depression, anxiety, fatigue, health-related quality of life or apathy^27,29,30^. However, as far as we know, this is the first study to assess the association between WMHs and ICDs in a sample of PD patients prior to undergoing DBS surgery (i.e. thorough examination suggested no comorbidities such as severe neuropsychiatric or major neurocognitive disorders).

Our retrospective analysis revealed that WMHs of presumed vascular origin were significantly correlated with the severity of ICDs in our younger patient group and the association was present even after controlling through a stepwise linear regression model for other possible factors influencing ICDs (Fig. 1), suggesting that WMHs are independently associated with ICDs. Although there could be several possible causes of WMHs seen on FLAIR images, an ischemic origin is frequently suggested by earlier pathology reports^31^. Some studies speculate that orthostatic hypotension in PD might contribute to the development of WM lesions^41,42^. Orthostatic hypotension is a common non-motor complication of PD occurring with a prevalence of about 30% (ranging from 9.6 to 64.9% across studies)^43^, and indeed, neurogenic orthostatic hypotension was found to be significantly associated with the severity of white matter lesions in PD patients^44^. However, it has remained unknown whether this relationship is causative or associative^44^ and it should be noted that several other potential factors, including oxidative stress or neuroinflammation, might also contribute to WM damage in PD^41^.

One of the most interesting findings of our study is the strong interaction effect between age and the severity of WMHs. We found that the significant positive association between lesion severity and ICDs was only present in younger patients, while a non-significant trend in the opposite direction (as demonstrated by negative Spearman’s rho values; Table 2) was found in older patients. We have no clear hypothesis for this finding, but there are several potential explanations. First, it is possible that the etiologies of WMHs are different between younger and older groups. However, answering this question is clearly outside the scope of our study; longitudinal and multimodal studies are required to examine this hypothesis. Previous studies have also suggested that younger age at PD onset is a key contributing factor of ICDs^1,12^, and age at PD diagnosis was clearly different between our younger and older groups. Although this group difference in age at onset did not result in a significant Total mMIDI score difference between our younger and older groups (Table 1), it is still possible that the etiologies of ICDs are different in patients diagnosed with PD (and thus receiving PD-related treatments) at older ages. Furthermore, it is possible that WMHs may predispose someone to certain ICD behaviors that are less frequent in older subjects. For example, gambling was present in 5 of our younger patients but none of our older patients (*p* = 0.054, Table 1). However, excluding these 5 subjects from our younger group did not change the positive association between the severity of WMHs and Total mMIDI score as indicated by Spearman’s correlations (*p* = 0.029, *p* = 0.023, *p* = 0.023, *p* = 0.009 and *p* = 0.009, respectively for PVWM-, DWM- and Total Fazekas scores, volume and number of WMHs). This argues against the above hypothesis but to answer this question properly, larger-scale future studies are needed that allow each ICD behavior to be examined separately. In addition, not only the severity of WMHs but also several clinical characteristics (e.g. DA LED, MDS-UPDRS scores, years of education) were interacted with age, which may suggest that the different correlation patterns of ICDs in the older subjects may be rather related to something more general than WMHs. Last, regarding dopaminergic treatment, it has also been presumed that antiparkinsonian medications may have different effects on different dopaminergic pathways. While dopaminergic medications may restore function of pathways responsible for movement, they may also potentiate the activity of mesolimbic pathways playing an important role in developing ICDs to a supraphysiological level^45^. It can be supposed that this supraphysiological stimulation of mesolimbic circuits may be more pronounced in younger subjects with higher residual dopaminergic functions and better compensating mechanisms. Future ICD studies in PD should carefully assess the presence of age-related interactions.

Previous studies suggested that ICDs or even atypical ICDs such as pathological generosity may be associated with brain damage, such as stroke, traumatic brain injury, and perinatal complications^46,47^. Here, we have demonstrated that even less malignant pathologies such as WMHs—which are often overlooked or generally considered as silent consequences of normal aging^31^—can be potentially associated with the presence of ICDs. The importance of WMHs regarding the non-motor symptoms of PD has been also supported by previous studies showing that changes in WMHs are positively associated with apathy, anxiety, fatigue or depression^27,29,30^.

Several previous studies tried to identify the risk factors for ICDs in PD and found a lot of potential contributors including demographic, disease-, treatment- and personality-related, addictive, cultural, psychiatric, and genetic factors among others^1,10,12,13^. However, at the same time, it is also acknowledged that ICDs are likely associated with specific structural and functional brain changes that may play a role in the diagnosis of ICDs in PD^15^. Based on our results, it appears that not only microscopic white matter changes, which require sophisticated methods for detection that are not usually part of clinical routine, but easy-to-assess macroscopic WMHs may also be associated with ICDs and might be an independent risk factor in PD patients. Future studies aimed at identifying risk and diagnostic factors for ICDs in PD should consider the severity of WMHs as a potential measure.

Of the disease-related factors, we found that MDS-UPDRS Total, mMDS-UPDRS Total, mMDS-UPDRS Part I, MDS-UPDRS Part I and Part II were most strongly associated with the Total mMIDI score in our younger PD group. The effect of MDS-UPDRS Part I on ICDs in PD has also been reported by others^48^, however, it should be noted, that Part I contains an item about the features of dopamine dysregulation syndrome (i.e. an ICD related behavior) that may be an underlying cause^34^. However, MDS-UPDRS Part I consists of several other components and if we calculated the modified versions of Total and Part I MDS-UPDRS scores by excluding this item (i.e. item 1.6), they still show significant correlations with ICDs (Table 2). Larger-scale future studies are needed to assess more carefully which items are behind the correlations between ICDs and MDS-UPDRS scores. However, it is also worth to realize that Total, Part I and Part II MDS-UPDRS, mMDS-UPDRS Part I and mMDS-UPDRS Total scores are highly intercorrelated with each other even if assessed in the whole patient group (n = 70, Spearman’s rho ≥ 0.703, *p* < 10^–6^) or separately in the younger (n = 35, rho ≥ 0.767, *p* < 10^–6^) or older (n = 35, rho ≥ 0.642, *p* ≤ 3·10^–5^) subgroups. Thus, it seems likely that not a single item, but rather an interplay of multiple factors are behind their correlations with ICDs.

In addition to MDS-UPDRS scores, known positive effects of DA LED, LEDD, anxiety and depression on ICDs were also identified when examining our whole patient population, similarly to previous studies^1,5,49^. Concentrating on our younger group, only the effect of DA LED remained significant, and using a stepwise linear regression model, it was shown that our results for WMHs were likely to be independent of this known factor.

### Methodological considerations

This was a retrospective study with a relatively small sample of PD patients from a single center who later underwent DBS surgery, thus selection bias may be considered. Most of our subjects had mild WMHs as indicated by a Total Fazekas score greater than zero but less than two. Further studies should be performed to assess the possible effects of more severe WMHs (i.e. Total Fazekas score ≥ 2) on ICDs. The imaging protocol was not fully standardized. However, correcting the final regression model for field strength and voxelsize did not change the significance pattern. Conventional routine 2D FLAIR images were used that may be less sensitive than 3D FLAIR and may allow less accurate assessment of WMHs. Only the global volume and number of WMHs were assessed. Future studies using more refined techniques such as 3D FLAIR combined with MPRAGE and/or DTI, may allow more precise and local (i.e. lobar or tract-specific) assessment of the WMHs and their impact on ICDs. The assessment of ICD severity was only based on Total mMIDI score. Other screening instruments were also developed for ICDs, such as Questionnaire for Impulsive-Compulsive Disorders in Parkinson’s Disease (QUIP) or its rating scale version (QUIP-RS)^1^ and easy-to-add-on methods were also suggested to identify ICDs more efficiently^8^. The lack of these additional screening tools (especially QUIP-RS) may be also considered as a limitation.

Our study was cross-sectional that limits understanding the causality between WMHs and ICDs. Although WMHs are probably not caused by ICDs, it may be also plausible that WMHs are not the main cause behind ICDs, however, a third factor (e.g. smoking) could be associated with both WMHs and ICDs. Comorbidities and other factors for premorbid personality may also be partly responsible for the correlations observed in our study. While it is known that smoking status is associated with both WMHs and ICDs^4,50^, there were only a few (i.e. n = 3) smokers among our younger patients (n = 35) and we had a patient with unknown smoking status. Therefore, the possible effects of smoking status on the association between WMHs and ICDs could not be statistically assessed in the present study. However, since 2 of the 3 smokers had no WMHs at all, it is unlikely that our results are driven by smoking. At the same time, an inherent limitation of our retrospective design that we have no data about the percentage of former smokers that would be also interesting to investigate in this context by future prospective studies.

Diabetes and cardiovascular diseases may also have an effect on the development of WMHs. Data about comorbidities such as diagnosed diabetes mellitus, hypertonia, ischemic heart disease and heart failure were available in all but one patients in our younger group either at the day of our examination (n = 13) or at a later timepoint (n = 21; minimum–maximum: 0.86–3.89 years later; mean: 2.43 years later; SD: 0.93 years). After excluding the single patient with no comorbidity data and all patients with any of the above comorbidities (known either at the time of the study or at a later time point), the final linear regression model resulted in the same significance pattern (n = 27, *p* = 1.1·10^–5^, *p* = 2.7·10^–4^ and *p* = 0.029 for the number of WMHs, MDS-UPDRS Part II and DA LED, respectively). This suggests that the association between WMHs and ICDs are not primarily driven by comorbidities. However, future studies including more patients with comorbidities may help better clarify this question.

## Conclusions

In conclusion, our pilot study supports the hypothesis that WMHs of presumed vascular origin may be independently associated with the severity of ICDs in younger patients with PD, providing further evidence that ICDs are not a simple side-effect of dopamine agonist therapy. Early recognition of ICDs in PD can be particularly important because in many cases, they are only recognized at a clinical level when they already have a serious impact on daily lives of patients (especially on their personal, financial or social statuses). Our results have the potential to improve early detection of ICDs in PD, however, further longitudinal and more sensitive studies are needed in the future to clarify the ability of WMHs in predicting ICDs.

## Data Availability

All relevant data are within the paper. Further data supporting the findings of this study are available from the corresponding author, upon reasonable request.
